# Neurohormonal Profiles of Assistance Dogs Compared to Pet Dogs: What Is the Impact of Different Lifestyles?

**DOI:** 10.3390/ani11092594

**Published:** 2021-09-03

**Authors:** Manuel Mengoli, Jessica L. Oliva, Tiago Mendonça, Camille Chabaud, Sana Arroub, Céline Lafont-Lecuelle, Alessandro Cozzi, Patrick Pageat, Cécile Bienboire-Frosini

**Affiliations:** 1 Research Institute in Semiochemistry and Applied Ethology (IRSEA), 84400 Apt, France; manuelmengoli@mac.com (M.M.); jessicaleeoliva@gmail.com (J.L.O.); t.mendonca@group-irsea.com (T.M.); c.chabaud@group-irsea.com (C.C.); s.arroub@group-irsea.com (S.A.); celine.lecuelle@sfr.fr (C.L.-L.); a.cozzi@group-irsea.com (A.C.); p.pageat@group-irsea.com (P.P.); 2 Clinical Ethology and Animal Welfare Centre (CECBA), 84400 Apt, France

**Keywords:** *Canis lupus familiaris*, guide dogs, biomarker, neuromodulator, serotonin, free oxytocin, total oxytocin, prolactin, stress

## Abstract

**Simple Summary:**

Dogs are currently involved in various roles in our society beyond companionship. The tasks humans assign to them impact their daily life and can sometimes create stressful situations, possibly jeopardizing their welfare. For example, assistance dogs need to manage their emotions in various challenging situations and environments. Thus, the capacity to cope with emotional stress is highly desirable in assistance dogs (~40% of assistance dogs fail to complete their education program). The emotional and stress responses are guided by brain processes involving neuromodulators. Neurohormonal profiling of these dogs can: (i) give cues about their emotional suitability to fulfill an assistance role; (ii) enhance their selection; and (iii) help to assess and improve their welfare state during the training course. We compared basal blood levels of three neuromodulators of interest between two populations, assistance vs. pet dogs. We found significantly different concentrations of oxytocin, a neuromodulator involved in social behavior. Levels of prolactin, a putative marker of chronic stress, were higher (although not statistically significant) and variable in assistance dogs. Dogs’ age also seemed to influence the various neuromodulators levels. These findings highlight the impact of different lifestyles undergone by dogs and the possibility to use neurohormonal profiling to monitor their effect on the dogs’ welfare and stress state.

**Abstract:**

Assistance dogs must manage stress efficiently because they are involved in challenging tasks. Their welfare is currently a fundamental issue. This preliminary study aimed to compare assistance dogs (AD; n = 22) with pet dogs (PD; n = 24), using blood neuromodulator indicators to help find biomarkers that can improve the AD breeding, selection, training, and welfare monitoring. Both populations originated from different breeds, are of different ages, and had different lifestyles. Basal peripheral concentrations of prolactin (PRL), serotonin (5-HT), free (fOT) and total (tOT) oxytocin were measured by immunoassays. Multiple linear regressions were performed to assess the effect of activity, age, sex, and their interactions on these parameters. Correlations between neurohormonal levels were analyzed. No interactions were significant. fOT and tOT concentrations were significantly influenced by age (*p* < 0.0001 and *p* = 0.0002, respectively) and dogs’ activity (*p* = 0.0006 and *p* = 0.0277, respectively). A tendency was observed for age effect on PRL (*p* = 0.0625) and 5-HT (*p* = 0.0548), as well as for sex effect on tOT (*p* = 0.0588). PRL concentrations were heterogenous among AD. fOT and tOT were significantly but weakly correlated (Pearson’s r = 0.34; *p* = 0.04). Blood prolactin, serotonin, and oxytocin may represent biomarkers to assess workload and chronic stress-related responses in ADs and eventually improve their selection and training.

## 1. Introduction

“Assistance dogs” (AD) include (i) service dogs; i.e., dogs that assist humans with mobility impairments as well as medical detection dogs and dogs assisting people with psychiatric impairments; (ii) hearing dogs; i.e., dogs that assist humans that have hearing impairments and (iii) guide dogs; i.e., dogs that assist humans that have visual impairments [1]. These dogs are often involved in complicated tasks (i.e., emotionally and cognitively challenging) in their daily life: they need to make the right behavioral choice while facing ambivalent situations, thereby adequately balancing their cognitive–emotional responses [2]. The term “ambivalent” refers to the notion of choice that can be equivocal. The assistance dogs must often choose between two situations, and sometimes go against their owner’s indications if necessary (e.g., in case of danger). For instance, the dog needs to show to not be over-reactive during walking, with good capabilities in filtering the external stimulation (social or environmental), being comfortable with and giving safety to the handler [3,4]. Good emotional balance and capacity to cope with emotional stress are therefore the most desired characteristics in trained guide dogs [5]. This is why dogs are often selected to be as calm as possible (“stability”/“passivity”), displaying no sign of aggression to other animals and humans (“docility”) and with a predisposition to demonstrate a good behavioral adaptation to their environment (“adaptability”). Conversely, distraction, shyness, and fearfulness are undesirable behavioral traits [6,7,8]. Breeds are often selected for assistance work on the basis of the degree of expression of these characteristics [9]. Individual characteristics will also influence the breeding and the educational protocols, with many dogs that fail, then are rejected or reoriented (to other assistance activities or offered for adoption) [7,10]: for instance, currently, the French guide dogs network face an approximate 40 percent loss of dogs due to physical or behavioral problems [11].

Only several research studies have investigated factors associated with long-term success in AD [2,7,8]. These dogs need to balance their emotional responses and try to find the best coping strategies to reduce their stress-related responses in a daily working routine. These emotional and stress responses are guided by brain processes involving neuromodulators. Investigating these neuromodulators in AD is an interesting approach to better understand their emotional and stress responses. Additionally, they can serve as indicators to help to assess the dogs’ individual emotional characteristics, hence their future chances of success in the AD programs. Among the neuromodulators which have an interest in the context of AD evaluation and follow-up, we focused on prolactin, serotonin, and oxytocin.

Prolactin (PRL) is a polypeptide neurohormone that plays multiple homeostatic roles in the organism in conjunction with the dopaminergic system [12]. PRL blood levels increase during acute or chronic stress (due to psychological or physiological stressors) and increased levels of PRL have been linked to emotional disorders and anxiety-related behaviors in several mammalian species [13,14,15]. PRL may change in response to psychological stress also in humans, where different studies have shown a positive correlation between anxiety and negative emotions and serum PRL [16,17,18]. Previous studies have indicated the interest in evaluating serum PRL in anxious dogs [19]. On the contrary, recent studies [20] highlighted that in sheltered castrated male dogs’ PRL concentrations correlate neither to stress scores nor to fear behaviors, and that a weak negative correlation between cortisol and prolactin levels was possible. In previous research, we found that AD presented higher levels of blood PRL than a pet dog population in a controlled situation [11,21], emphasizing the interest of further investigating this neuromodulator.

Serotonin or 5-hydroxytryptamine (5-HT) is a monoamine found in the central nervous system, gastrointestinal tract, and blood. It has broad physiological functions as a neuromodulator [22]. Serotonergic neurons have numerous projections throughout the brain, provide 5-HT to the rest of the central nervous system, and can modulate limbic-HPA activity [23]. 5-HT was demonstrated to be an interesting neuromodulator to study dog behavioral and emotional responses since low levels of 5-HT were found to be associated with animal distress and behavioral disorders, such as impulsiveness, loss of motor control, and aggression in certain dogs [24,25]. It was shown that aggressive dogs had significantly lower serum concentrations of 5-HT than non-aggressive dogs [24,26]. For AD, intraspecific and interspecific aggression are unacceptable behaviors, that are unequivocally associated with dogs’ rejections, with the animal exiting the educational program and the school [6,11]. Therefore, we found it crucial to assess this neuromodulator in AD as a part of the dog’s emotional evaluation before and during their educational training. 

Oxytocin (OT) is a nonapeptide produced by the hypothalamus and released from magnocellular neurons that project to the posterior lobe of the pituitary where it is naturally secreted into the bloodstream and acts as a hormone, mainly involved in parturition and lactation [27]. OT is also transported to various brain regions via axonal pathways where it acts as a neuromodulator, with a crucial role in many social behaviors, ranging from social memory and affiliation (sexual and parental behaviors, attachment, pair-bonding) to aggression (“mate-guarding,” maternal aggression) (for reviews, see [28,29,30,31]). In particular, OT has been described as being involved in positive social intra-species interactions [32,33,34,35] and inter-species interactions, such as human-animal interactions [36,37,38,39,40]. In dog applied ethology, OT is often related to research on how a social interspecific interaction with humans [41,42] or social stress [43] will modify OT levels, or on the effect of OT external administration on dogs’ cognition and communication and other behaviors or aptitudes [21,44,45,46,47,48]. For instance, intranasally applied oxytocin has been shown to increase positive expectations [44], increase the use of human social cues such as pointing [48], modulate the dogs’ aggressive response to the threatening cues of a human [49], increase affiliation toward owners and conspecifics [38], increase intraspecific play behaviors [34], and influence the visual contact with the owners [45,46]. AD need to show a predisposition for positive prosocial interaction with humans, good mental flexibility, and being smartness, with an important role of OT in all these aptitudes [41,46,50].

Currently, OT has become a renowned neuromodulator, an indicator commonly investigated in behavioral and neuroendocrinological research, in particular, human–dog interactions [42,51]. Several studies have reported a coordinated release of central and peripheral OT [52,53] and showed that peripheral levels can provide a minimally invasive indicator of central state [54], from a practical point of view, when central parameters are not available. In dogs, OT concentrations have been peripherally quantified in plasma, urine, and saliva [55,56,57] despite important methodological considerations and caveats. OT is difficult to assay and methods to measure endogenous OT greatly vary [55,58]. This results in a lack of correlation between them and in discrepant results among the literature, hence these methods are still under development [59]. MacLean et al. [59] proposed that OT molecules may exist under different molecular conformational states, such that these different states may be differently measured by the various used methods, leading to the observed discrepancies. Among the putative molecular states of OT, Brandtzaeg et al. [60] developed a method assaying the protein-bound OT in plasma. OT is circulating in two forms, the most known free OT (fOT), and the protein-bound form, both fractions constituting the total amount of OT (tOT) in plasma. Different authors are working to describe different methods to measure total OT levels and protein-bound levels and to find what their biological functions may be [50,61,62]. Measuring only the fOT, as currently recommended [63], may, in many cases, be a confusing factor, since the fOT concentration can change because of dynamic intrinsic or extrinsic factors or substances that displace OT from proteins [58,64]. Furthermore, Uvnäs Moberg et al. [65] recently hypothesized that OT may be a principal hormone and exert its effects through its active fragments, adding more consideration to the possible importance of the different OT molecular forms.

The aims of this study were (i) to describe the neurohormonal profiles of two different populations of dogs (assistance dogs: AD and pet dogs: PD) displaying different daily routines and lifestyles in relation to their breed selection, by measuring their basal peripheral neuromodulator levels that may be of importance regarding the monitoring of their socio-emotional behaviors i.e., PRL, 5-HT, and OT; (ii) to further investigate the biological significance of the different molecular states of oxytocin by exploring both total and free oxytocin (tOT and fOT) basal levels in these two different populations of dogs; (iii) to look at the putative associations between these neuromodulators peripheral measures, considering their physiological interactions within the central nervous system [66,67].

We expect that the different lifestyles experienced by the AD and PD as well as the AD individual and breed selection may result in substantial differences in their basal levels of these circulating neurohormones involved in social behavior regulation. Beyond that, we thought establishing the neurohormonal profile of AD may help assess their future chance of success such that AD selection before entering the educational program may be refined. That is particularly important considering the percent of failures and the cost of this loss [11,21]. In addition, using neurohormonal profiling to evaluate their emotional responses to the workload and challenging situations they face during their training may help assess their welfare. This may conduct to an improvement of the educational programs and hopefully, in the longer term, to establish guidelines about the training of AD.

## 2. Materials and Methods

### 2.1. Subjects

The dog population involved was the same previously presented in Oliva et al. [21]: fifty-one dogs were involved at the beginning of the trial and checked by a veterinary behaviorist to look for possible medical or behavioral reasons to exclude them. Five dogs were excluded because 1 blood drawing did not provide a sufficient sample’s volume, 1 was discovered pregnant, 2 showed a phobic response to humans and possible aggression, and 1 had moved during the trials. The final sample, therefore, included 46 dogs that were blood sampled, considering 22 young AD (9 Males and 13 Females) with a mean age of 1.10 ± 0.17 years and 24 PD (16 Males and 8 Females) with a mean age of 4.98 ± 3.13 years. The dogs did not present unhealthy conditions, and they were not under any medication or showing any behavioral or emotional disorders (as shown by the use of the validated emotional disorders evaluation scale (EDED), see [21]). When females were entire, they were tested at least 2 months out of estrus.

All AD came from the Frédéric Gaillanne Foundation (FGF; L’Isle-sur-la-Sorgue, France), an AD training school where they were in training to be guide dogs or dogs involved in assistance of children with autism spectrum disorder. All dogs recruited from the FGF were in training and still living part time with the same human caregiver who had been assisting the FGF to raise them since they were between 8 and 10 weeks old (“foster families”). FGF dogs entered the school at about 12 months, and they were involved in a specific training for at least one and half years, from Monday to Friday at the school, excluding the weekends and the holidays, when they were with their foster families. They were following the same diet and performed similar exercise and daily activities, proportionally to their age. All dogs came from the same breeding selection: they were Labrador Retriever or Saint-Pierre Dogs (a crossed breed of Bernese Mountain Dogs and Labrador Retriever) (Table 1).

PD were a heterogeneous clinical population. All animals were older than 10 months, of different breeds (Table 1), sizes, following different diets, and with different daily routines. They were recruited from owners that heard about the trial due to publicity on social media and social networks; other animals were referred by other clinics. Hence, the two dog populations of this study underwent a different daily routine in terms of environmental stimulation, richness of intra- and interspecific social interactions, and mental and physical training.

### 2.2. Ethical Statement

This study was performed in accordance with French (2013-118) and European law (2010/63/EU) on the protection of animals used for scientific purposes. The study was approved by the IRSEA Ethics Committee C2EA125 (approval number AFCE_201605_02).

### 2.3. Blood Sampling and Processing

All dogs were accommodated in the same consultation room of the CECBA (Clinical Ethology and Animal Welfare Centre) facilities (Apt, France), with the same environmental temperature (21–23 °C) at the same time schedule. Moreover, all owners were asked to keep their dogs indoors the night before the blood drawing to minimize the effect of ambient temperature on neurohormones’ secretion.

With the help of an operator, blood was taken by a veterinarian from the cephalic vein or the jugular vein in the least stressful conditions possible and only if the immobilization was feasible. An amount of 8–10 mL of total blood per dog was collected into pre-chilled pink EDTA-Aprotinin vacuum tubes (BD^®^ tubes, Elvetec, Pusignan, France) to collect plasma and approximately 1 mL was collected into red vacuum tubes with gel separator (Vacuette^®^ Greiner Bio-One, Alcyon, Paris, France) to collect serum. The pink tubes were immediately transferred to an ice compartment where they remained until centrifugation, while red tubes remained at room temperature for between 30- and 180-min. Samples were centrifuged at 1600× *g* for 15 min at 4 °C. Serum and plasma were transferred to a plastic tube and stored at −20 °C until analyses. Because of the necessity to differently concentrate and process the plasma/serum for the various analyses, certain samples were of insufficient volume to be assayed for each parameter. Then, the number of final samples immunoassayed and used for the statistical protocols was different regarding the neuromodulators (PRL: 21 AD and 24 PD; 5-HT: 22 AD and 24 PD; fOT: 20 AD and 21 PD; tOT: 21 AD and 19 PD).

### 2.4. Neurohormones Analyses

Neurohormone analyses were performed by the IRSEA labs (Research Institute in Semiochemistry and Applied Ethology, Apt, France).

PRL concentrations from canine serum were measured using the canine Prolactin ELISA kit (#DEV9944, Demeditec Diagnostics^®^, Kiel, Germany), according to the manufacturer’s instructions. The lower detection limit for canine prolactin was 0.4 ng/mL (assay range: 0–80 ng/mL).

Serum 5-HT levels were measured by enzyme immunoassay (Serotonin ELISA kit #ADI-900-175, EnzoLifeSciences, Villeurbanne, France) according to the manufacturer’s instructions and the validated method detailed in the referred application note [68]. This kit had already been used in previous studies assaying 5-HT in a variety of animal species, including the dog [69]. The kit’s sensitivity is 0.293 ng/mL with an assay range between 0.49 and 500 ng/mL.

Plasma fOT was assayed using the Oxytocin ELISA kit from Cayman Chemical (#500440, Ann Arbor, MA, USA) according to the manufacturer recommendations, including the plasma solid-phase extraction on C18 columns (Hypersep 1 g, Thermo Fisher Scientific, Illkirch, France), allowing a two-times concentration factor, as explained in [21]. Plasma tOT was assayed using the same ELISA kit after reduction/alkylation and protein precipitation (R/A PPT) procedures, which release bound OT from plasma proteins and allow for the detection of much higher concentrations of OT in dog plasma, according to the previously published methods [60,61]. Chemicals used in this procedure (acetonitrile, dithiothreitol, iodoacetamide) were provided by Sigma-Aldrich (Saint-Quentin-Fallavier, France).

### 2.5. Statistical Analysis

All data were analyzed in SAS 9.4 software (Copyright (©) 2002–2012 by SAS Institute Inc., Cary, NC, USA). The significance threshold was classically fixed at 5% for all the following statistical analyses. First, preliminary statistical tests were performed to evaluate the effect of the dog’s activity (PD vs. AD) on age and sex and determinate if these last should be included in the statistical analysis. For the age variable, comparison between the two groups (AD and PD) was performed with the Wilcoxon/Mann–Whitney test with the NPAR1WAY procedure. Conditions of normality and heteroscedasticity were tested respectively with the UNIVARIATE and TTEST procedures and have not been checked. For sex and activity effects, their association was studied with the Chi-Square test with the FREQ procedure.

Then, for PRL, 5-HT, fOT, and tOT, a multiple linear regression was performed using the GLM procedure. Effects of activity (AD and PD), sex (male and female), age, and the three corresponding interactions were tested in the complete model. Conditions of normality of residues and homoscedasticity were tested using respectively the UNIVARIATE and the GPLOT procedures. For the age effect (quantitative variable), the linearity assumption was verified with the GPLOT procedure. For fOT and tOT, these conditions were verified. For PRL and 5-HT, normality of residues was not verified; a Box-Cox transformation was applied to data using the TRANSREG procedure, which allowed to verify the normality and then the homoscedasticity. The three interactions were removed of the model because they were not significant.

The correlation between fOT and tOT in both dog populations was assessed using Pearson correlation analyses with the CORR procedure since both sets of data were normal. The correlations between 5-HT, fOT and PRL were evaluated using Spearman correlation analyses due to the CORR procedure since the normality of data was not verified.

## 3. Results

Blood concentrations of PRL, 5-HT, fOT, and tOT in dogs are shown in Table 2.

### 3.1. Preliminary Statistical Analyses to Assess the Similarity between AD and PD Groups Regarding the Age and Sex Factors

A significant difference was observed between the two groups (AD and PD) for age (Wilcoxon/Mann–Whitney, N = 46, Z = −5.0057, *p* < 0.0001) (AD: mean ± SD = 1.10 ± 0.17 years versus PD: mean ± SD = 4.98 ± 3.13 years). No significant difference is observed between the two groups for sex (Chi-Square, N = 46, DF = 1, χ^2^ = 3.0693, *p* = 0.0798) (female: 13 AD versus 8 PD and male: 9 AD versus 16 PD).

### 3.2. Multiple Linear Regressions

**Figure 1:**
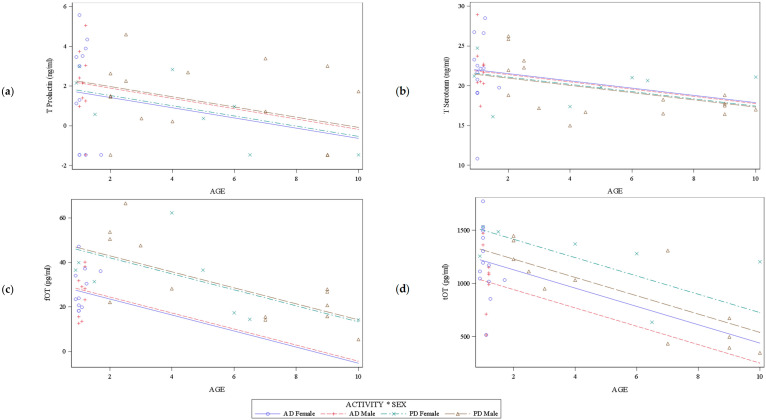
Linear regression models of T (Transformed) prolactin (**a**), T (transformed) serotonin (**b**), free oxytocin (fOT) (**c**), and total (tOT) (**d**) according to age, activity, and sex. Individual values are marked with points of different shapes and the regression lines are shown in solid or broken lines of different colors according to the legend. The parallel regression lines highlight that there is no interaction between the factors. Merged or closed regression lines indicate that there is no effect of the factors’ activity/sex, or not much difference between them, respectively. Identical slopes of the regression lines denote similar effects of the age, regardless of the dogs’ activity/sex.

Only the age factor appeared to be significantly different between the AD and PD group. However, we observed a tendency for the sex factor. Then, we decided to perform a multiple linear regression analysis including the three factors: activity (AD vs. PD), sex (M vs. F), age (as a quantitative variable) for each of the four parameters (PRL, 5-HT, fOT, tOT). We also investigated the interactions between these three factors (Table 3). None was significant in the complete model. Then, we could remove them from the complete model to focus on each of the three factors independently (simplified model). Figure 1 displays graphical representations of the linear models obtained for each parameter according to the three factors tested (Age, Sex, and Activity).

In all panels, we clearly observed the linear relationship between the neurohormonal parameters and age for each activity and sex group. We could also remark the greater dispersion of points around the regression line for PD due the inequal repartition of dogs according to age, with AD being significantly younger (less than 2 years old), as previously stated.

Detailed results of the significant statistical models are shown in Table 4.

Activity, age, or sex had no significant effect on PRL levels that is shown by the close regression lines for sex and activity observed in Figure 1a. Only a tendency was observed for the factor age (Table 3), with older dogs tending to display lower levels of PRL (β = −0.2570; SE = 0.1342), as also displayed by the negative weak slopes of the regression lines in Figure 1a. Nevertheless, looking at the descriptive data (Table 2), AD displayed mean and median serum PRL levels at least two times higher than PD. We noticed an important heterogeneity of serum PRL concentrations among the AD compared to PD, with a standard deviation (SD) of 18.6 vs. 8.6 ng/mL, respectively, and a larger range of concentrations in AD than in PD, due to the different maximum values reached in each population: 71.3 ng/mL in AD vs. 38.8 ng/mL in PD. The Fisher test assessing the equality of variances gave the following outcomes: F-value = 4.73, *p* = 0.0005, confirming that variances are heterogenous within both populations.

Similarly, there was no significant effect of the activity, age, or sex on 5-HT peripheral concentrations, as showed by the merged regression lines in Figure 1b. However, a substantial tendency appeared for the factor age (Table 3): the older the dogs were, the lower the 5-HT levels tended to be (β = −0.4573; SE = 0.2315), also revealed by the negative weak slopes of the regression lines in Figure 1b.

Both factors activity and age had significant effects on the fOT concentrations. Age appeared to be a confounding factor since the older the dogs, the lower the fOT (*p* < 0.0001) (Table 4). Thus, for each additional year of age of the dog, the fOT level decreased by an average of 3.59 pg/mL, regardless of the activity of the dog. After adjustment for age, fOT levels were on average 18.52 pg/mL lower in AD than in PD (*p* = 0.0006). This effect of activity was also denoted by the distance between the regression lines of each activity group in Figure 1c. Conversely, the regression lines of both sexes for the same activity group were merged, indicating no sex effect, in accordance with the insignificant *p values* of the simplified model shown in Table 3.

Similarly, both activity and age had significant effects on tOT concentrations and the age appeared to be a confounding factor too: the older the dogs, the lower the tOT (*p* = 0.0002) (Table 4). Thus, for each additional year of age of the dog, the tOT level decreased by an average of 86.52 pg/mL, regardless of the activity of the dog. After adjustment for age, tOT levels were on average 285.82 pg/mL lower in AD than in PD (*p* = 0.0277). Regarding the sex effect (Table 3), there was also a tendency on tOT concentrations: females tended to display higher tOT levels than males (β = 187.1804; SE = 95.9044). These effects are also suggested by the distance between the four regression lines observed in Figure 1d.

There was a significant correlation between fOT and tOT levels in the entire dog population, which was positive but weak (N = 37; Pearson’s r = 0.34; *p* = 0.04). The correlation between 5-HT and PRL was not significant (N = 45; Spearman’s Rho = 0.157; *p* = 0.30). The correlation between 5-HT and fOT showed a tendency and was positive and weak (N = 41; Spearman’s Rho = 0.290; *p* = 0.07). Finally, the correlation between fOT and PRL was not significant (N = 41; Spearman’s Rho = −0.014; *p* = 0.93).

## 4. Discussion

In this study, we aimed to describe basal blood levels of certain neuromodulators involved in socio-emotional behaviors i.e., PRL, 5-HT, and OT (free and total) in AD vs. PD (with AD having different ages, different daily routines and being of different breeds) and to explore their putative relationships. We found that fOT and tOT concentrations were significantly influenced by the dogs’ age and activity (as PD or AD): younger dogs had higher levels of fOT and tOT, whereas ADs had lower levels of fOT and tOT. As the AD were significantly younger than PD, both age and activity effects were confounded, thus explaining why the simple observation of descriptive data did not allow to show these outcomes. We also found tendencies on the effect of sex on tOT concentrations (higher in female) and the effect of age on PRL and 5-HT concentrations, following the same direction: the younger the dogs are, the higher their levels of peripheral neurohormones are. We also observed that peripheral PRL levels are heterogeneous in the population of AD compared to PD, which is the expression of a greater variability of this parameter within AD population. Additionally, this study showed that fOT and tOT plasma measures are significantly but weakly correlated, with an association of only 11.6%. Other neurohormonal parameters were not significantly correlated between them.

### 4.1. Prolactin

Median serum PRL levels found in AD and PD can be considered as normal according to the reference values provided by the ELISA manufacturer user’s guide and matched those described in the literature for normal dogs [70,71]. In this study, there were no significant differences in serum PRL between AD and PD, although mean and median PRL concentrations were at least 2-times higher in AD than in PD. A precedent study [21] with the same population involved in a different protocol (sub-divided in three groups receiving different treatments and undergoing several blood samplings) showed the average prolactinemia was significantly higher in AD vs. PD, while here, we only considered the basal level of serum prolactin to reflect the basal state of these two populations. Importantly, the age effect was not considered in [21]. We found here that the age tended to influence the blood PRL levels with younger dogs displaying higher PRL basal concentrations. This new statistical approach on the same dog population could in fact explain the previous results as well as the descriptive data we observed here on AD: the higher PRL levels might be due to the younger age of AD rather than their activity. Further studies comparing AD and PD of the same age can help to clarify this question. Alternatively, the higher prolactinemia, we also observed in descriptive data of AD, could be related to breed/genetic characteristics, as suggested by previous literature [72,73]. AD are notably selected on desirable behavioral characteristics (meaning that only animals which do not show aggressiveness, chronic phobias, or anxiety are kept for reproduction purposes), which can originate from the presence of genetic elements involved in the neuromodulation of behaviors/emotional responses. In turn, these genetic characteristics can be associated with the dog’s breeds, which are more commonly selected for AD recruitment.

This higher prolactinemia observed from descriptive data may also be linked to a chronic-stress response: serum PRL concentrations have already been associated with emotional disorders in dog [19]. Because of the inhibitory control of dopamine on PRL secretion [74], PRL represents an interesting biomarker in behavioral medicine that may reflect the dopaminergic system activity, previously described as correlated with different anxious states in dogs [19,75]. Usefully, the levels of blood PRL are more stable than those of dopamine, strongly influenced by its more variable clearance features [76], thus making PRL easier to analyze. Thus, investigating the dopaminergic system activity via the measurement of blood PRL can help assess the chronic-stress responses and anxiety in dogs and follow-up their welfare states. This is of particular interest in AD who must face substantial workload and challenging situations in their daily life. However, the exact influence of age and breed on PRL levels should be determined before using it as a reliable biomarker of stress and emotional responses.

The important heterogeneity of serum PRL concentrations among the AD compared to PD is also noteworthy. PD were dogs come from different litters with no possibility of genetic familiarity, with different ages, sizes, breeds and with a different daily routine, whereas the AD population represents a population of relatives from a genetics point of view, with the same diet, size and with a similar daily routine (the same training plan and daily life at the school). Thus, PRL unexpectedly seemed to represent a more variable parameter in the AD population than in the PD population of this study. This possible individual variability within a homogenous dog population should also be considered to examine PRL as a biomarker.

### 4.2. Serotonin

In our study, there was no significant effect of dog’s activity on 5-HT levels, whereas dogs’ age tended to have an influence on it. This may partly explain the apparent difference between mean peripheral 5-HT concentrations of AD and PD that we can observe in the descriptive data (Table 2) since AD are significantly younger than PD. This may represent an interesting feature especially for young AD in training, facing workload and challenging situations since 5-HT has patience-promoting properties and is related to coping and stress moderation [77,78], implying that their welfare may be preserved in this training context [79]. However, this age-effect tendency was not found in previous studies [80,81,82]. Conversely, breed, diet, environment, exercise, and housing seemed to influence 5-HT concentrations in dog serum according to the literature [25,69,83]. Authors have shown considerable interbreed variation in canine serum 5-HT concentrations [25,81,82]. The population of AD we included in this study all belong to Labrador-related breeds. Therefore, breed and selection features may also fully or partly explain our descriptive data observations.

The serum 5-HT concentrations found in PD were similar to those reported by previous studies [25,69,82] but are higher than those described by others [79,80,81]. These differences can arise from the variable characteristics (other than the breed and the age) of the dog population used in each study: shelter dogs, obese dogs, dogs with valvular diseases, etc. They can also arise from different preanalytical factors and analytical procedures used among these studies [80]. For instance, the antibodies’ affinity and specificity are crucial features in antibody-based assays, and they can differ across the available ELISA commercial kits, resulting in measurement variability. Here, we used an ELISA kit previously validated for the use in dogs’ serum, following the application note of the manufacturer [68].

Despite the fact that peripheral findings may not reflect central serotonergic activity, as highlighted by Alberghina et al. [79], serum 5-HT is still considered an accessible model to study the response in the central serotonin system [84], especially in dogs considering the simplicity of the methodology [24]. Alberghina et al. [79] demonstrated a weak positive relationship between levels of serum 5-HT and sociability of dogs toward human (in terms of approaching, handling, playing). The latter is of utmost importance for AD population and their training. Behavioral characteristics such as handedness, ability to remain focused, a good emotional balance and positive prosocial behaviors are fundamental in AD and the subjects not presenting these behaviors are always reoriented or rejected [11], including eliminated from reproduction for breeding. Because of its link with coping and docility vs. aggressive behaviors, 5-HT remains an interesting neurohormonal biomarker for AD follow-up.

### 4.3. Free and Total Oxytocin

In our study, age significantly influenced plasma fOT and tOT levels. After adjustment for age, plasma fOT and tOT concentrations were also found to be significantly different according to dogs’ activity as AD or PD, with lower levels of fOT and tOT in AD than in PD. This was unexpected since OT has often been associated with sociability, friendliness, and docility toward humans in many species, particularly in assistance animals [38,56,85]. The OT system was suggested to be involved in the dog domestication process [86,87], including if a recent study in dogs and wolves showed that life experience was more influential on OT levels than domestication, showing a positive correlation between the physical contact with the owners and the OT concentrations [88]. The AD involved in our study have a different lifestyles and life experience compared to PD; they undergo repeated separations from their foster family (that fostered them during their first year of age and then on the weekend during their training) every Monday as part of their 6–8 months-long training; from Monday to Friday, they are trained and performed different activities/exercises during the day, and at night they stay in a kennel at the guide school. This particular life experience may impact the attachment processes of AD and influence the neuromodulatory system involved in bonding, i.e., the OT system. This may explain the lower levels of fOT in AD that we found in this study. In addition, the influence of breeds (especially the particular AD breeds) on fOT and tOT basal levels is unknown and deserves further investigations.

In another population of AD, McLean et al. [42] described comparable plasma concentrations of fOT (around 19 pg/mL) before human-animal interactions. In another experiment [61], the same authors compared OT concentrations between PD and a population of AD that have been bred for affiliative and non-aggressive temperaments and found that AD had higher free and total OT, findings that were not replicated in our study. Conversely, once the age was adjusted, we found here that AD displayed lower levels of fOT and tOT. Examining the results, it appears that our population of PD displayed higher fOT concentrations than in MacLean et al.’s study, this may be due to an intrinsic population variability of PD (see [64] for more information about the effects of individual factors on the peripheral OT). Their populations of AD and PD also seemed to differ regarding the age factor (with PD and AD having a mean ages similar than in our study, approximately 4.9 years and 1.6 years respectively). Thus, it might be possible that this age difference also influenced the levels of fOT and tOT they found in the AD population compared to the PD population. We also found higher levels of tOT than in their study; despite the use of similar plasma preparation methodologies (reduction/alkylation and protein precipitation), we used different commercial assay kits (Arbor Assay #K048 vs. Cayman Chemical #500440). The variability between antibodies among OT immunoassays may explain the difference observed between the tOT levels we found and those found by MacLean et al. [61], as later hypothesized by other authors [59].

In addition, the factor sex tended to have an effect on plasma tOT concentrations in our study. This was not the case in the previously cited study of MacLean et al. [61]. However, in a previous study, we found a sex effect on tOT in horses [50], but in the opposite direction (higher tOT concentrations on males than in females). In the same study, we also investigated the relationship between fOT and tOT fractions in conjunction with the sex and lifestyles/social activities in dogs and horses, and it could differ among species [50]. In dogs, a weak correlation seems to exist between fOT and tOT, an aspect that will be interesting to further investigate to facilitate the laboratory analyses and steps of sample collection and preparation [55,59]. However, further research is needed to clarify the role of tOT, from a biological point of view, before considering possible research or clinical applications [50,60]. All these observations reinforce the interest to investigate possible neuroendocrine predictors of individual differences in social behavior and their critical roles in shaping dog social behavior, including aspects of both affiliation and aggression [42,61]. Furthermore, it is important to consider the role of OT in prosocial positive interactions and bonds with humans, be they owners, foster families, dog trainers or animal keepers [41,48,51].

### 4.4. Neuromodulators Correlations

Finally, no correlation between blood 5-HT, fOT (known to be the biological active form [58]) and PRL was statistically present. As such, it seems prudent to analyze the results independently. However, these results revolved around peripheral outcomes and then must be assessed for what they are: they do not dismiss central interactions between these neuromodulators. For instance, it was shown that PRL presents an important relationship with central OT that regulates PRL’s secretion in many social behaviors [66]. Similarly, previous research demonstrated the link between the serotoninergic and oxytocinergic systems in the brain [67,89,90,91].

### 4.5. Implications and Perspectives

Research is ongoing to understand the causes of AD’s reform or reorientation, highlighting the importance to focus on long-term factors in dogs’ success [2,7,8] in order to avoid as much financial and time losses as possible because of reformed/reoriented AD, considering the high cost of each dog’s education and life-care (e.g., possible total cost of 20,000–25,000 euros per dog in France [11]). It is then important to better profile behavioral tendencies and AD emotional and stress responses to increase dog welfare and to be able to detect possible distress during working activities and daily routines [92]. Monitoring basal blood levels of relevant biomarkers in AD may help to reach these objectives and eventually improve the selection of AD, as well as their breeding, by ameliorating the choice of the most “emotionally competent” individuals, as based only on their behavior. To the best of our knowledge, this study is the first to describe and profile different basal biomarkers involved in different neuromodulation systems of emotional and stress responses i.e., blood PRL, 5-HT, fOT and tOT in PD vs. AD that have different daily routines and social activities. Age was found to be a significant confounding factor for fOT and tOT. A similar tendency was observed for PRL and 5-HT. OT, which modulates bonding, social attachment, and social behavior was found to be significantly different between AD and PD, after age adjustment, with lower levels in AD that in PD. This observation may be connected to the particular life experience (repeated separations form the main attachment figure, the foster family) undergone by AD during their training.

This study also underlined the high impact of within-population variability on the other neuromodulators’ levels, which emphasizes the need of an individual profiling over time, to be able to detect meaningful variations and undesirable emotional or distress responses in each AD dog due to its particular training/daily life. The lack of correlation between the blood levels of these neuromodulators requires independent investigation of each of them to be able to draw an overall emotional profile. Ideally, an individual follow-up during all their careers of AD will allow to highlight if their neurohormonal profiles can be associated with their future success and if this profiling can be helpful in predicting it. Eventually, neurohormonal profiling may help improve the selection of smarter ADs as well as the assessment of their educational program and workload to guarantee the best possible success of the AD training.

The low correlation between fOT and tOT, as well as the biological meaningfulness of tOT warrants further investigation. Other neuromodulators may also show differences between AD and PD, and it would be interesting to explore these deeper in the future. In particular, vasopressin, the neuropeptide closely related to OT and also involved in a wide range of social behaviors, including aggression, and stress responses is certainly of high interest in this context [61]. Finally, it may also be beneficial to apply such approaches to other working dogs. Further studies improving our knowledge about their physiology, neuroendocrinology, and behavior, due to meaningful biomarkers to support desirable temperament/behavior and breed selection, will be surely welcomed to improve the training procedures and dogs’ welfare.

## 5. Conclusions

In conclusion, this study established neurohormonal profiles of dogs (AD and PD) in order to give cues about their emotional and stress responses and coping abilities. This can be especially beneficial in the context of working dogs, such as assistance dogs in this study, to (i) improve their selection based on their inherent neurobiological features and avoid time and money loss by educating and training unsuitable dogs; and (ii) assess the suitability of their training program and their resulting lifestyle by evaluating their impact on dogs’ stress, emotions, and welfare via the measurement of neurohormones linked to these processes. This study showed that AD can display different levels of various neurohormones than PD, particularly fOT and tOT, which are of high interest for AD, considering its involvement in bonding processes and social behaviors. To a lesser extent, PRL levels may also differ, and this should be further investigated, considering its association with emotional disorders. Finally, this study also emphasized the age influence on basal neurohormonal levels among dog populations. Thus, individual monitoring of neuromodulators over time, allowing to control for sex and age variability, seems to be preferable to properly evaluate each dog’s emotional responses.

We hope this work can pave the way to a new approach based on individual neurohormones profiling/monitoring to assess the welfare states of dogs involved in animal-assisted activities and, in the end, improve their selection/training for better chances of success.

## Figures and Tables

**Table 1:** Breed differences between assistance dogs (AD) and pet dogs (PD).

Origin of Dogs			
Pet Dogs (PD)		Assistance Dogs (AD)	
Breed	N	Breed	N
Mixed	3	Bernese Mountain dog × Labrador (St Pierre)	15
Border Collie	3	Labrador	6
Welsh Corgi Cardigan	2	Labrador × golden retriever	1
Australian Shepard	2		
German Shepard	2		
Labrador	2		
Border Collie Cross	1		
Australian Shepard × German Shepard	1		
Bernese Mountain Dog	1		
Labrador × Boxer	1		
Malinois	1		
Samoyed	1		
Poodle	1		
Yorkshire Terrier	1		
Westie	1		
Pinscher × Chihuahua	1		

Adapted from Oliva et al. [21].

**Table 2:** Descriptive data for basal peripheral concentrations levels of prolactin (PRL), serotonin (5-HT), free and total oxytocin (fOT and tOT) for assistance dogs (AD) and pet dogs (PD).

Dogs	Neurohormones	Mean	SD	Median	Min	Max	Lower Quartile	Upper Quartile
AD	PRL (ng/mL)	14.2	18.6	6.8	0.2	71.3	2.5	18.7
	5-HT (ng/mL)	1220.3	615.9	1114.5	144.6	2664.7	876.6	1339.4
	fOT (pg/mL)	27.1	9.7	26.0	12.7	47.2	19.1	35.1
	tOT (pg/mL)	1136.7	320.7	1113.6	517.1	1774.1	1018.7	1362.1
PD	PRL (ng/mL)	6.3	8.6	3.2	0.2	38.8	0.7	10.0
	5-HT (ng/mL)	868.5	450.1	702.1	359.2	1958.3	525.8	1064.5
	fOT (pg/mL)	30.8	17.2	27.8	5.4	66.5	15.8	39.9
	tOT (pg/mL)	1032.4	405.4	1206.3	347.7	1535.6	635.6	1371.6

SD: standard deviation.

**Table 3:** Results of the multiple linear regressions for PRL, 5-HT, fOT and tOT variables including activity, age, and sex effects and their interactions.

Variable	Complete Model			Simplified Model		
	Interaction	F Value	*p*-Value	Factor	F Value	*p*-Value
PRL	Activity × Sex	0.20	0.6566	Activity	0.02	0.9014
	Age × Activity	1.00	0.3245	Age	3.67	0.0625
	Sex x Age	0.71	0.4045	Sex	0.53	0.4707
5-HT	Activity × Sex	0.40	0.5330	Activity	0.10	0.7518
	Age × Activity	0.00	0.9799	Age	3.90	0.0548
	Sex × Age	1.95	0.1703	Sex	0.01	0.9058
fOT	Activity × Sex	1.33	0.2564	Activity	13.96	0.0006
	Age × Activity	2.17	0.1497	Age	19.27	<0.0001
	Sex × Age	0.49	0.4896	Sex	0.05	0.8160
tOT	Activity × Sex	0.18	0.6776	Activity	5.27	0.0277
	Age × Activity	1.93	0.1737	Age	16.85	0.0002
	Sex × Age	2.01	0.1658	Sex	3.81	0.0588

**Table 4:** Unstandardized regression coefficients for each predictor having significant effects following the multiple linear regression analyses for fOT and tOT variables.

Variable	Predictor	β	SE	*p*-Value
**fOT**	Activity (Reference PD)	−18.5189	4.9556	0.0006
	Age	−3.5911	0.8180	<0.0001
**tOT**	Activity (Reference PD)	−285.8182	124.5404	0.0277
	Age	−86.5206	21.0776	0.0002

β: unstandardized regression coefficients or estimates; SE: standard error; PD: pet dogs.

## Data Availability

The data presented in this study are available on request from the corresponding author.
